# Predictors of subclinical carotid atherosclerosis in middle-aged women

**DOI:** 10.1371/journal.pone.0197582

**Published:** 2018-05-23

**Authors:** Isly L. de Barros, Laura Costa, Bento Bezerra, Rafael Gomes, Natanael Morais, Célia M. C. Strunz, Moacir Novaes, Otávio C. E. Gebara, Rodrigo Pinto Pedrosa, José C. Nicolau

**Affiliations:** 1 Instituto do Coracao (InCor), Hospital das Clinicas HCFMUSP, Faculdade de Medicina, Universidade de Sao Paulo, São Paulo, Brasil; 2 Hospital Universitário Oswaldo Cruz (HUOC)–University of Pernambuco, Recife, Brazil; 3 Pronto Socorro Cardiológico de Pernambuco (PROCAPE)–University of Pernambuco, Recife, Brazil; 4 Centro Integrado de Saúde Amaury de Medeiros (CISAM)–University of Pernambuco, Recife, Brazil; 5 Cardiology Department–Hospital Santa Paula, São Paulo, Brazil; University of Bologna, ITALY

## Abstract

**Background:**

Traditional strategies for primary cardiovascular prevention have been insufficient in reducing the high rates of coronary ischemic events in women, probably because these women are often stratified into low-risk groups. However, cardiovascular diseases continue to be the main cause of morbidity and mortality in women worldwide. We hypothesized that carotid atherosclerosis (CA) is common in middle-aged women.

**Methods:**

We prospectively evaluated asymptomatic peri- and post-menopausal women with no cardiovascular diseases or the use of hormone therapy from two gynecologic clinics. All the patients underwent full clinical and laboratory evaluation and underwent a B-mode ultrasound for carotid evaluations. The presence of CA was defined as the presence of plaque and/or carotid intima-media thickness (CIMT)>1.00 mm. We performed logistic regression to evaluate independent predictors of CA.

**Results:**

We studied 823 women (age: 54.4±5.4 years; body mass index-BMI: 28.5±4.9 kg/m^2^; diabetes:10%; hypertension: 58%). The prevalence of CA was 12.7% for the entire population and 11% for the low-risk sub-group as defined by a Framingham risk score <5%. In the multivariate model, age: odds ratio (OR) = 1.54, 95% confidence interval (CI) = 1.25–1.89,p<0.001; current smoker status: OR = 2.69, 95% CI = 1.48–4.91, p = 0.001; total cholesterol: OR = 1.13, 95% CI = 1.03–1.24, p = 0.008; and systolic blood pressure: OR = 1.01, 95% CI = 1.00–1.02, p = 0.030 remained independently associated with CA.

**Conclusion:**

Subclinical CA is common among asymptomatic middle-aged women, and traditional risk factors are independently associated with CA. These findings are particularly relevant for improving cardiovascular health in women.

## Introduction

The risk of cardiovascular disease (CVD) in women has been underestimated over the years, particularly due to the misconception that women are protected against CVDs[1]. However, CVDs continue to be the main cause of morbidity and mortality in women worldwide, particularly in middle-aged women[2,3];middle age is a period characterized by age-related changes in reduced estrogen production and an increase in cardiovascular risk[4]. Menopause marks a clear shift in women’s risk profiles for CVDs, and atherosclerosis is the main cause of morbidity and mortality starting at menopause. Furthermore, there are many gaps in the understanding of ischemic heart disease in women, both in terms of its clinical aspects and diagnostic procedures, making early detection of vascular disease in this population difficult[5]. Therefore, preventive interventions are highly desirable.

Measurement of the carotid intima-media thickness (CIMT) is a non-invasive technique for quantifying the extent of subclinical atherosclerosis and predicting the risk of CVD events[6]. Moreover, the presence of atherosclerotic plaques also correlates with the future risk of cardiovascular events, regardless of CIMT[7].

Women are under-represented in the cardiology literature, and as a consequence of the scarcity of data related to females, CVD prevention and treatment recommendations for women are based predominantly on evidence derived from studies that only or predominantly enrolled men[1]. Therefore, the main purpose of this study was to assess the prevalence and risk factors associated with subclinical carotid atherosclerosis (CA) in middle-aged women.

## Methods

### Study design and population

In this cross-sectional study, 1315 consecutive women aged 45–65 years with irregular or interrupted cyclic menses in the previous year were prospectively recruited at two gynecology outpatient clinics from October 2009 to October 2011. The protocol was approved by the ethics committees of the involved institutions (University of Pernambucoand Instituto do Coracao-InCor, Hospital das Clinicas HCFMUSP, Faculdade de Medicina, Universidade de Sao Paulo, Brasil) and was conducted in accordance with local regulations. Written informed consent was obtained from each subject.

### Sample size estimation

The sample size was calculated using the following parameters: (a) expected accuracy level of the results, (*e*) = 0.035; (b) level of sample confidence set at 95%; (c) expected proportion (*P*_*e*_), p = 0.5 (the population’s expected proportion was unknown, and we opted to adopt the maximum variability); and (d) *z-*value, the value of the normal curve related to the 95% confidence interval (CI). Finally, we calculated a sample size of 800 women.

### Clinical assessments

The data were uniformly collected and transcribed according to a research protocol. Women were classified according to the criteria of the Stages of Reproductive Aging Workshop (Straw) Report as follows: (a) early pre-menopausal or peri-menopausal status as determined by an irregular menstrual cycle with intervals longer than 60 days and shorter than 365 days or follicle-stimulating hormone (FSH) <35 mIU/mL or (b) late peri-menopausal or post-menopausal status as determined by ≥365 days between menstrual cycles or FSH ≥35 mIU/mL[8]. Blood pressure (BP) was measured using the oscillometric technique with an arm digital sphygmomanometer. At least three BP measurements were taken at one-minute intervals, and the average between the two final measurements was considered the individual’s BP. Systolic BP over 140 mm Hg and diastolic BP over 90 mm Hg or those on antihypertensive drugs were considered hypertensive[9]. Diabetes was defined as a fasting glucose >125 mg/dL or the use of hypoglycemic drugs[10]. Framingham sex-specific risk equations (FRS) were used to predict the risk of developing coronary disease events (myocardial infarction or coronary heart disease death) over the next 10 years[11].

### Biochemical assessment

Blood samples were collected after a fasting period of 12 hours for biochemical testing. Venous blood was collected from all the participants to measure fasting glucose, total cholesterol, low-density lipoprotein, high-density lipoprotein and triglycerides. For FSH measurements, the electro-chemiluminescence immunoassay was utilized. The quantitative determination of high-sensitivity C-reactive protein was performed through immunonephelometry. The aldosterone and adiponectin levels were quantified using a radioimmunoassay technique.

### Carotid ultrasound measurements

A 12-3-MHz EnVisor Ultrasound System (Philips Ultrasound, Bothell, WA-USA) was used to evaluate the carotids. All image acquisitions were performed by a single professional. The far wall common CIMT and the presence of plaques were defined according to the Mannheim Carotid Intima-Media Thickness and Plaque Consensus[12]. CA was identified by the presence of CIMT>1 mm and/or the presence of plaques. The measurements of CIMT were obtained at the far wall on the left and right of the carotid common artery (CCA). The data were recorded and subsequently analyzed using a semi-automated edge-detection software package that provided an index of success based on the quality of the acquired images. The CIMT measurement was defined as a composite measure that combined the maximum IMT common carotid of the left and the right carotid arteries and the arithmetic average of these measurements. A plaque was defined as follows: a) a parietal structure thicker than 1.5 mm, b) luminal protrusion >0.5 mm, or c) thickness >1.5 times the adjacent CIMT.

### Statistical analyses

Continuous variables are described as the means ± standard deviations or medians (interquartile intervals) according to a normal distribution after performing the Kolmogorov-Smirnov test. To test for possible associations between CA and other variables, univariate and multivariate analyses were conducted. For univariate analyses, Student’s *t*-test (normal distribution) and the Mann-Whitney test (non-normal distribution) were used. For the analysis of categorical variables, the chi-square test was utilized. For the adjusted models, the stepwise logistic regression test was used with CA as the dependent variable. All the variables that were correlated with CA in the univariate analyses with a p-value <0.20 were initially included in the model as independent variables. Two-tailed p-values <0.05 were considered statistically significant, and the STATA 9.0 software program (StataCorp. 2005. *Stata Statistical Software*: *Release 9*. College Station, TX, USA: StataCorp LP) was used for the statistical calculations.

## Results

**Fig 1:**
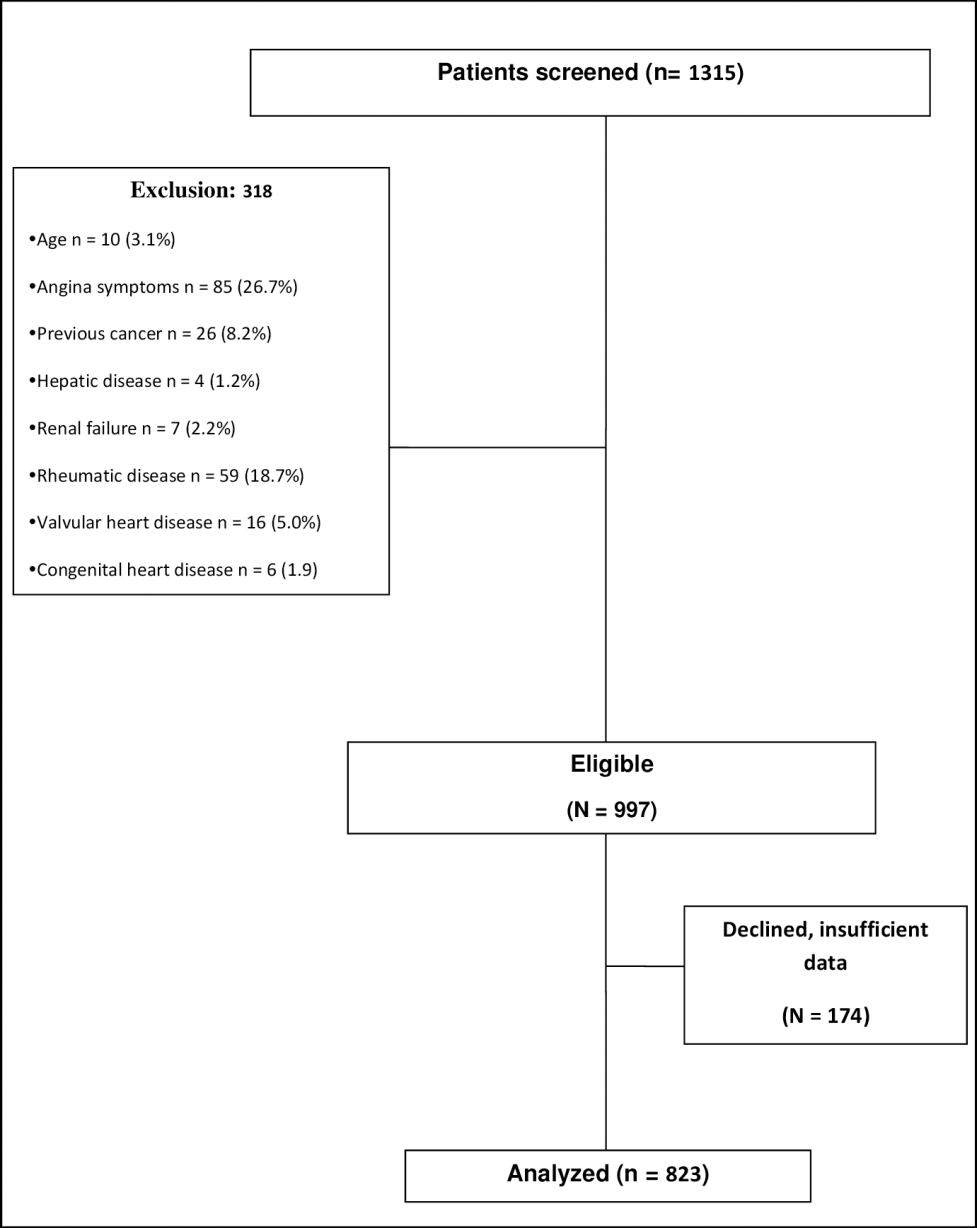
Flow diagram of the study population.

From the initially screened population of 1315 women, 318 did not fulfill the inclusion criteria, while 174 women refused to participate or were excluded due to insufficient data, resulting in a final sample of 823 participants. The flow diagram of the study population is shown in Fig 1.

The anthropometric and clinical characteristics of all the patients, as well as the patients categorized according to CA status, are summarized in Table 1. The population consisted of predominantly middle-aged and overweight women. The frequency of hypertension and diabetes were 58% and 10%, respectively, and most of the participants (77%) were post-menopausal. When compared to participants without CA, those with CA were older, had a higher frequency of hypertension and higher blood pressure, and were more likely to be a smoker. A greater proportion of these patients were in the high-risk category for FRS. Women with CA had higher total cholesterol and LDL-C levels than women without CA. No significant differences were identified between the groups with or without CA in regard to fruit or vegetable consumption or laboratory measures such as hsCRP, adiponectin, and aldosterone (Table 2).

**Table 1:** Associations between carotid atherosclerosis and socio-demographic, lifestyle and clinical characteristics.

Variables				
	Total	CA (+)	CA (-)	p
Women number, n	823	105	718	-
Age, years^a^	54.4 ± 5.4	56.6 ± 5.2	54.1 ± 5.4	< 0.001
Skin color, (n, %)^b^				
White	251(30%)	32 (30%)	219 (31%)	-
Mulata	406 (49%)	59 (56%)	374 (52%)	0.728
Black	139 (174%)	14 (13%)	125 (17%)	0.433
Income, (n, %)^b^				
> 2 wages	133(16%)	22 (21%)	111 (15%)	-
≥ 1 and < 2 wages	364(44%)	43 (41%)	321 (45%)	0.955
< 1 wages	134(16%)	20 (19%)	114 (16%)	0.717
**Lifestyle**^b^				
Sedentary	233(28%)	34 (32%)	199 (28%)	0.977
Current smokers, (n,%)	84 (10%)	18 (17%)	66 (9%)	0.014
No Fruit / vegetable intake / day, (n, %)	48 (6%)	7 (7%)	41 (6%)	0.845
**Medical history**^b^				
Family dx CHD (n, %)	308 (37%)	46 (44%)	262 (36%)	0.156
Hypertension (n, %)	477 (58%)	76 (72%)	401 (48%)	0.002
Diabetes (n, %)	81(10%)	15 (2%)	66 (8%)	0.113
Depressed (n, %)	172 (21%)	22 (14%)	150 (56%)	0.791
**Climacteric variables**^b^				
Post-menopausal	634 (77%)	86 (82%)	548 (76%)	0.222
Hot flashes (n, %)	485 (59%)	57 (54%)	428 (60%)	0.286
**FRS**^b^				
< 5%	666 (81%)	73 (70%)	593 (83%)	-
6–10%	49 (6%)	9 (9%)	40 (6%)	0.121
>10%	107 (13%)	23 (22%)	84 (12%)	0.003
**Physical characteristics**^a^				
BMI, kg/m^2^	28.5 ± 4.9	28.3 ± 4.9	28.5 ± 4.9	0.647
Waist circ., cm	91 ± 11	91 ± 11	91 ± 11	0.581
Systolic BP, mm Hg	128 ± 20	133 ± 19	127 ± 20	0.003
Diastolic BP, mm Hg	83 ± 12	85 ± 12	83 ± 12	0.304

^**a**^Continuous variables are presented as the means ± SDs.

^**b**^Nominal variables are presented as absolute numbers and percentages.

Abbreviations: CA: Carotid atherosclerosis, dx CHD: diagnosis of coronary heart disease, FRS: Framingham Risk Score, BMI: body mass index, Circ: circumference, BP: blood pressure.

**Table 2:** Associations between carotid atherosclerosis and laboratory variables.

Variables				
	Total	CA (+)	CA (-)	p
Fasting glucose, mg/dL^a^	99±37	107 ± 51	99 ± 33	0.054
TC, mg/dL^a^	215 ±45	230 ± 44	214 ± 43	0.001
HDL-C, mg/dL^a^	53±23	51 ± 11	53 ± 11	0.119
LDL-C, mg/dL^a^	142±42	154 ± 41	140 ± 41	0.001
TG, mg/dL^a^	137 ±84	152 ± 89	137 ± 93	0.127
CRP, mg/dL^b^	0.19 (0.09–0.41)	0.21 (0.11–0.43)	0.16 (0.08–0.39)	0.736
FSH, mIU/mL^b^	61 (40–82)	59 (39–82)	61 (40–82)	0.888
Adiponectin, μg/ml^b^	9.1 (6.4–12.7)	6.8 (4.2–9.5)	6.0 (3.7–9.0)	0.336
Aldosterone, ng/dL^b^	6.1 (3.8–9.1)	6.6 (4.0–9.5)	6.1 (3.8–9.1)	0.980

^**a**^Continuous variables are presented as the means ± SDs.

^**b**^Variables with skewed distribution are presented as median (interquartile range).

Abbreviations: CA: Carotid atherosclerosis, TC: total cholesterol, HDL-C: high-density lipoprotein cholesterol, LDL-C: low-density cholesterol, TG: triglycerides, hsCRP: high-sensitivity C-reactive protein, FSH: follicle-stimulating hormone.

As shown in Table 3, CA was detected in 105 women, or 12.7% (95% confidence interval [CI] 10.5–15.0%) of the study population. When analyzed separately, increased CIMT (>1 mm) and carotid plaques were detected in 14 (1.7%) and 99 (12.0%) subjects, respectively. The prevalence of CA increased to 30.3% (95%CI 27.1–33.4%) if a CIMT value ≥75^th^ percentile was used as the threshold. Univariate analyses revealed an association between CA and each of the following: age, current smoking status, history of hypertension, systolic blood pressure, total cholesterol, and LDL cholesterol. In the multivariate model, age: odds ratio (OR) = 1.54, 95% CI = 1.25–1.89, p<0.001; current smoker status: OR = 2.69, 95% CI = 1.48–4.91, p = 0.001; total cholesterol: OR = 1.13, 95% CI = 1.03–1.24, p = 0.008; and systolic blood pressure: OR = 1.01, 95% CI-1.00–1.02, p = 0.030 remained independently associated with CA (Table 4).

**Table 3:** Prevalence of carotid atherosclerosis, abnormal CIMT, and carotid plaque.

	N = 823		
Prevalence	N	%	95% CI
CA	105	12.7	10.5–15.0
CIMT > 1.0 mm	14	1.7	0.8–2.59
Carotid plaque	99	12.0	9.8–14.3

Abbreviations: CA: Carotid atherosclerosis, CIMT: intima-media thickness of the common carotid artery.

**Table 4:** Associations of risk factors with carotid atherosclerosis by multivariable analyses (adjusted model).

Variables	Univariate OR (95% CI)	p	Multivariable OR (95% CI)	p
Age, years	1.53 (1.26–1.86)^a^	< 0.001	1.54 (1.25–1.89)^a^	< 0.001
Current smokers	2.04 (1.16–3.59)	0.014	2.69 (1.48–4.91)	0.001
SBP, mm Hg	1.015 (1.01–1.02)	0.003	1.01 (1.00–1.02)	0.030
Glucose, mg/dL	1.11 (1.00–1.24)	0.054	1.09 (0.97–1.23) ^b^	0.125^b^
TC, mg/dL	1.16 (1.05–1.27)	0.001	1.13 (1.03–1.24) ^b^	0.008^b^

^a^Chance with each variation of 5 years in age

^b^chance with each variation of 20 units.

Legends: SBP: systolic blood pressure TC: total cholesterol.

## Discussion

This study evaluated CA among consecutive middle-aged women and found several important findings: we demonstrated a high prevalence of subclinical CA. The overall prevalence was 12.7% when we considered a CIMT>1 mm and/or the presence of plaque and 30.3% (95%CI 27.1–33.4%) if a CIMT value ≥75th percentile was used. As expected, we found a significant association between the CA frequency with age and some traditional risk factors for atherosclerosis, such as smoking, cholesterol and systolic blood pressure.

In regards to the prevalence of CA in females, using a definition similar to ours, Prati et al. reported a higher percentage (26.4%) than that obtained in this study[13]. However, these researchers analyzed a general population that was older than the population included in the present study, which may explain the observed differences. Because the population included in our study consisted of asymptomatic women and a large majority (80.8%) was classified as having very low risk (<5%) according to the FRS, a low prevalence of CA would be expected. Contrary to this expectation, the prevalence of CA was 12.7% for the entire study population and 11% for the very low-risk population, as determined by the FRS, primarily at the cost of plaque presence. These findings are of particular relevance as strategies for reducing cardiovascular risk are based on risk prediction models in which younger women are often classified as having low cardiovascular risk. As a consequence, this population is often not included in cardiovascular prevention programs[14,15].

We found a mean CIMT of 0.645±0.124 mm in the entire study population. A broad comparison with previous studies is difficult, primarily due to the different protocols and definitions of CIMT. Trémolliereset al.[16] found smaller mean values for the CIMT (0.523±0.067 mm) in comparison with our findings. However, our population presented a higher frequency of cardiovascular risk factors, a finding that could explain the differences in CIMT values. By contrast, in two studies in the Chinese population, Yu et al.[17] reported a mean CIMT of 0.76±0.12 mm in post-menopausal women, and Sun et al.[18] analyzed 1781 asymptomatic Chinese individuals (of whom 650 were women) and found a mean CIMT of 0.66±0.11 mm. Finally, in a Latin America multicenter study, a mean of 0.65 mm (0.60–0.74 mm) was reported, which was very similar to our findings[19].

From the prognostic point of view, in the ARIC (Atherosclerosis Risk in Communities) study, the incidence of cardiovascular events adjusted by age, race, and the study location was greater among those with CIMTs≥1.00 mm and was more noticeable among women (hazard ratio [HR] = 5.07, 95% CI 3.08–8.36) than that among men (HR = 1.85, 95%CI 1.28–2.69)[20]. Similarly, a recent meta-analysis by van der Oord et al. showed that for every one standard deviation increase in the CIMT, the risk of myocardial infarction increased by 26%, and the risk of stroke increased by 31%[21].

The carotid plaque frequency was 12% in our study, and only 1.7% of the women showed CIMTs>1.0 mm. When we set the reference CIMT value to the ≥75^th^ percentile, the prevalence of CA increased to 30.3% (95%CI 27.1–33.4). In a population of post-menopausal asymptomatic Chinese women, the carotid plaque prevalence was 21.8%[17], specifically 25% and 54% in pre- and post-menopausal women, respectively, as reported in the WHLP (Women’s Healthy Lifestyle Project) and the HWS (Healthy Women Study) reports[22]. However, among middle-aged French women, the carotid plaque prevalence was 8.1%, using a reference CIMT value of ≥1.75 mm as the definition of a plaque[23]. Taking into account the definition utilized in the present study, the CARMELA study showed a prevalence that ranged from 5% (Mexico City) to 14% (Barquisimeto)[17]. Compared to CIMT, the presence of atherosclerotic plaques is a better predictor of events[21]. This observation lends strength to our findings, in which the presence of CA was primarily related to the presence of plaque.

From the traditional risk factors that were analyzed, only smoking, total cholesterol, and SBP demonstrated independent associations with the presence of CA in our study. Previous publications have shown similar results: Sutton-Tyrrel et al.[22] analyzed the WHLP and HWS populations and reported that SBP and smoking were associated with the presence of carotid plaque in pre-menopausal women and that in post-menopausal women, a significant association was identified between carotid plaque and smoking, pulse pressure, and age. In a post-menopausal Chinese population, Yu et al.[17] found a significant association between the presence of carotid plaques and age, waist-hip ratio, and LDL-C. In regards to diet, Mattioli et al. [24] showed a lower incidence of asymptomatic atherosclerosis in a population of pre-menopausal women who had greater adherence to the Mediterranean diet. However, a meta-analysis by Hartleyet al. [25] reported limited evidence, and very few studies recommended an increase in fruit and vegetable consumption in the absence of additional dietary interventions or lifestyle changes. More trials are needed to confirm this finding.

The present study has some strengths and limitations. The strength of our study includes the recruitment of consecutive women from the gynecology outpatient clinics, which generalizes our findings. However, as patients were provided by the government social security and from low-income households, the social demographics may influence our findings even though we did not see a difference between the groups with and without CA. Furthermore, our findings are derived from a cross-sectional study, and we cannot infer causality but, rather, only an association between CA with cardiovascular risk factors.

## Conclusion

In conclusion, our study revealed a high prevalence of CA among asymptomatic middle-aged women. As expected, traditional risk factors were independently associated with CA in this population. Therefore, efforts to increase cardiovascular prevention programs among women are warranted.
